# Topics of Public Interest

**Published:** 1907-08

**Authors:** 


### TOPICS OF PUBLIC INTEREST.

### Medical Education in the United States.

**With Some Facts of the Existing Conditions and Needs as Shown in the Report of the Council on Education Made to the American Medical Association at Atlantic City, June 4-7, 1907.**

[*By the Council of Medical Education.*]

DURING the last twenty-five years medicine has made wonderful progress. During this period it has earned for itself the right to be called a science.

The science of medicine is one of the broadest sciences. It is based on the sciences of anatomy and physiology, physics and chemistry, pathology and bacteriology and pharmacology. For centuries medicine was a mass of empirical facts which acknowledged no limitations and the acceptance of which required a robust faith on the part of both doctor and patient. Today medicine is a science; it knows its power, it recognises its limitations and has learned to acknowledge without fear those things which it does not know, and when it makes such acknowledgment it turns the fire and zeal of well-trained minds on these unsolved problems, with the hope and determination of finding their solution.

The benefits conferred on mankind by medical discoveries have already been enormous.

The recognition of the germ cause and resulting control by quarantine of great plagues like cholera, yellow fever and pest, the virtual eradication of smallpox by vaccination, the cure of diphtheria by antitoxin, the relief from pain secured by anesthetics, the prevention of surgical infections by Lister's great discovery, antiseptic surgery, which has made possible the many life-saving operations of modern surgery, the reduction of typhoid fever by a pure water supply—all these have added greatly to the wealth and health of communities and to the happiness and comfort of the individual.

The civilised world can well afford to acknowledge the importance of the accomplishments of modern medicine and to extend to medicine every encouragement and assistance in solving the many problems still in sight.

The cancer problem, tuberculosis—"the great white plague"—pneumonia—the captain of the men of death—scarlet fever, with its undiminished mortality; all these and many more must be conquered and controlled. And in this fight the community must furnish the laboratories and hospitals necessary and train the men who are needed to carry on this struggle of science against disease.

In our own country medicine has so far received little assistance from the state, and, compared with schools of liberal arts and theology, it has received almost no endowments from individuals. And yet no state and no philanthropist can find a better investment than the hospital and laboratories of a modern medical school; none from which the immediate returns are so large, none from which the possibilities of enormous profits to humanity are so great.

Modern medicine requires a better order of intellect and better training than it did twenty-five years ago, and better than that possessed by the average student entering its ranks today in this country.

The standards of medical education in the United States are very uneven, representing the highest and the lowest types as compared with such powers as England, France and Germany. As a whole, the standard in this country is distinctly lower than in these countries and lower than it should be to meet the requirements of medical science in the present stage of development.

In this country the control of medical education and licensure is vested in the individual states and not in the national government. This has probably been for the best because of the enormous size of our country and the widely varying conditions met in the different sections.

In this country, until comparatively recently, medical education has been in the hands of private medical colleges, conducted by groups of medical men largely for their own interests. In Germany and France the medical school has been developed as a department of the university, and, fortunately, in this country the same plan is being gradually adopted. It must be said to the credit of American medical schools and American medical men that, handicapped as they have been, without state aid or private endowment and dependent practically entirely on the fees of students, they have accomplished much, of which we may justly be proud.

### BRITISH MEDICAL EDUCATION.

In England medical education is in control of the national government through the agency of the Medical Council. The Medical Council determines the standard of preliminary education, the character of the curriculum, the length of the course and the character of the examinations for licensure.

The preliminary education required is about equal to our best four-year high schools and this is followed by a five years' course in medicine, the first year devoted largely to physics, chemistry and biology, and this year can be taken either in the medical school or in a school of liberal arts recognised by the council. Then follow the four years of medicine, given largely as they are in this country; the last year, however, can be taken as a clinical year in a recognised hospital. The examination for licensure can then be taken at the end of this five-year course or it can be taken in two parts, one after the completion of the laboratory years and the second on the completion of the course. As a matter of fact, however, these examinations are so rigid, that the average time required by the student to prepare for them is about six years.

### MEDICAL EDUCATION IN GERMANY.

In Germany the student can enter the medical department of a university on leaving the gymnasium or a scientific school. The medical course is now about six years divided as follows: The first year is devoted to physics, chemistry and biology; then follows a four-year course such as is given in our better schools, and then a sixth year, which must be spent as an interne in a hospital. At the end of this time the student can come up for his state examination or it can be taken as in England in two parts, one part after he has finished his laboratory studies, anatomy, physiology, etc., and one after the completion of his clinical work.

The conditions of medical education in this country are not satisfactory. There are too many medical schools. The preliminary education demanded is often insufficient. Many medical schools are conducted purely as business ventures and give an unsatisfactory course, have poor facilities, lack trained teachers, and, besides, graduate a large proportion of men who fail before the comparatively simple and fair examinations required by the state examining boards for a license to practise medicine.

The Council on Medical Education of the American Medical Association, a committee of five composed of Prof. Councilman of Harvard, Prof. Frazier of the University of Pennsylvania, Prof. Vaughan of Michigan, Prof. Witherspoon of the Vanderbilt University, and Prof. Bevan of Rush Medical College, has been studying this question for the last three years. During the last year members of this council made a personal inspection of the medical schools of the United States in order to determine the existing conditions of medical education in this country.

Each school was visited by some member of this committee and was marked as an individual taking a civil service examination, on the character of its facilities for teaching modern medicine and its actual work and the results of its teaching as shown by the success or failure of its graduates who come before the state examining boards for a license to practise medicine.

The schools were divided according to their standing into three groups: Group I, in which were placed all the schools above 70, which was taken as a passing mark. Group II, which included the schools marked from 50 to 70. This mark was regarded as not acceptable, but it was considered that the deficiencies in this group might be remedied by such improvements as would bring the standing above 70. Group III, in which the markings were below 50, the facilities being entirely inadequate and the work bad.

In this country there are 160 medical schools, about as many as in Great Britain, Germany, France, Austria, Belgium, Holland, Denmark, Greece, Hungary, Italy, Norway, Sweden, Portugal, Roumania, Russia, Spain and Switzerland combined. Great improvements have been made in medical education in this country during the last few years. Until recently, within twenty-five years, almost all the schools in America gave a full medical course in two years. Now all the schools demand a four-year course.

MEDICAL SCHOOLS IN EUROPE.

| | |
|---|---|
| Austria | 7 |
| Belgium | 4 |
| Denmark | 1 |
| Great Britain and Ireland | 43 |
| France | 16 |
| Germany | 21 |
| Greece | 1 |
| Hungary | 3 |
| Italy | 20 |
| Netherlands | 4 |
| Norway | 1 |
| Portugal | 2 |
| Roumania | 2 |
| European Russia | 12 |
| Spain | 8 |
| Sweden | 3 |
| Switzerland | 5 |
| Total | 153 |

The above figures are approximately correct and are based on the report of the Commissioner of Education, as well as on the report in the book called "Minerva," a yearbook of libraries, colleges and universities, published by Dr. Karl Trübner, Strassburg. It may be said, therefore, that there are in the United States as many medical schools as in entire Europe.

Many schools have increased their requirement for admission and laboratory and hospital facilities, until they can now offer as good medical instruction as can be obtained anywhere in the world. A splendid movement to bring up the requirements of the preliminary education of the American medical student to that of the European standard has been started, and about fifty schools have agreed to require, in addition to a four-year high school course, one year or more of physics, chemistry and biology by the year 1910.

THE STANDARD.

In spite of the advances made and good work done by a considerable number of American medical schools, the general average is extremely low. Modern medicine demands a good preliminary preparation and thorough technical training. The standard which should be ultimately generally required is the following: 1, A four-year high school education; 2, one year of chemistry, physics and biology; 3, two years in well-equipped laboratories of anatomy, physiology, pathology and pharmacology; 4, two years in clinical work in dispensaries and hospitals, and, 5, one year as an interne in a hospital.

The average student would leave high school at 18 years and graduate at 24 years of age, and today it is impossible to acquire a sufficient knowledge of medicine with less preparation. The schools in this country represent all grades from the very highest —the best of our schools are as good as the best in the world—to the very lowest, a large number being little better than diploma mills.

Of the 160 schools only about 50 per cent. are sufficiently well equipped to teach modern medicine. About 30 per cent. are doing poor work and need to make great improvements in their facilities and character of instruction to bring them to an acceptable standard, and about 20 per cent. have no claim to recognition whatever.

It was clearly shown by this inspection that many medical schools are conducted for profit, and that the medical schools conducted solely for the profit of their faculties are a menace to the community and the profession.

There are four night schools teaching medicine in the United States, three in Chicago and one in Philadelphia. It is evident that modern medicine which requires four, five or six years of hard study and work, to which the student must devote the entire day and part of the night, can not be mastered in the night schools between the hours of 7 and 10 p.m., especially when the student has devoted the rest of the day to some other occupation. Many of the poor schools are conducted as quiz classes for the purpose of preparing the student to pass the state board examination and not with the object of making him a competent practitioner.

### THE REMEDY.

How can this unsatisfactory condition of medical education be remedied? Two things are necessary, proper state control, and financial support. It is in the power of the public and the profession to enact and enforce laws which will secure proper standards of medical efficiency. A state without the protection of good medical laws, well enforced, becomes the dumping ground for poorly-prepared medical men. The state examining boards are unfortunately in some states merely political machines.

In this country we need money for medical education. It costs more to educate a student than he can pay in the way of fees. Medical education must secure state aid and private endowment. No better investment can be made by any state than that put into medical research and education. The public must be taught the present condition and the necessities of modern medicine, and philanthropists must be shown that medicine well deserves the support that is given schools of liberal arts, theology, libraries, and the like. It is the duty of the profession to secure a high standard of preparation and efficiency of the men who are legally qualified to practise medicine.

### THE NEED OF COOPERATION.

The public has a right to demand such state control as will insure its protection against ignorance and inefficiency and will secure for it the services of well-qualified practitioners when they apply for such services, and the public should insist on the legislation necessary to secure such protection.

In the effort to secure higher standards the entire medical profession of America, without regard to the so-called schools of practice, should unite in one common movement to secure the needed legislation and reforms. No attempt should be made by legislation to force the regularly licensed medical attendant on individuals who believe in mental, religious and other means of healing, except in diseases such as diphtheria and smallpox, which are in the control of the state and municipal boards of health and in which the police power is exercised to protect the entire community and applies equally to all classes.

If the public realised the enormous difference that exists between well-trained modern medical service and ignorant, inefficient medical service, they would soon demand and obtain the needed reforms.

The present conditions of medical education and qualifications in this country are not satisfactory. These facts can easily be determined by any one qualified to investigate the subject and should be widely known both to the profession and to the public. Without any sensationalism the profession, the public and the press should be enlisted in the effort to secure the needed state control and financial assistance, without which it will be impossible to bring medical education and service up to the desired standard of efficiency.

In this country of great wealth and great population and of high average intelligence we can no longer be satisfied with our present standards of medical education, which are so much below those of Germany, France and England. Nor should we be satisfied with any except the highest and best.

### SUMMARY.

In brief, the situation of medical education in the United States may be given as follows:

(*a*) A three years' careful study has been made by the Council on Medical Education of the American Medical Association of the conditions surrounding medical education in the United States. This study included the inspection of all the schools in the United States by one or more members of the Council.

(*b*) The great advance in the sciences in recent years has created the necessity for a much broader and more thorough education, both preliminary and medical, for the physician equipped to practise modern medicine.

(*c*) The standards of the medical schools in the United States are very uneven, representing the highest and the lowest types as compared with the standards of England, France and Germany. As a whole, the standard in this country is unsatisfactory and much lower than in those countries.

(*d*) A modern medical education demands, 1, a four-year high school education; 2, a year of physics, chemistry and biology; 3, two years in well-equipped laboratories of anatomy, physiology, pathology and pharmacology; 4, two years in clinical work in dispensaries and hospitals; 5, a year as interne in a hospital.

(*e*) The expense for the equipment and maintenance of the modern medical school is greater than can be met by fees paid by medical students. Medical schools, therefore, need endowments in order to meet the demands of present day medicine.

(*f*) In the United States, until recent years, medical education was mostly in the hands of medical colleges conducted as private institutions, while in Europe it is controlled by the universities. Within recent years, however, some of the medical colleges in this country have secured university connection.

(*g*) There are still, however, a large number of schools which are conducted solely for profit, and profit is only possible where the college fails to provide proper facilities for laboratory and clinical training.

(*h*) There are 160 medical schools in the United States alone, as many or more than there are in all the countries of Europe combined. Of the 160 medical schools in the United States only about 50 per cent. are sufficiently equipped to teach modern medicine, 30 per cent. are doing poor work and need to make great improvements, while about 20 per cent. are unworthy of recognition.

(*i*) If the public realised the enormous difference that exists between well-trained modern medical service and ignorant inefficient medical service they would soon demand and obtain the needed reforms.

(*j*) A state without the protection of good medical laws, well enforced, becomes the dumping ground of the low-grade medical school with its output of illy-prepared medical men.

(*k*) To secure better conditions requires two things: Endowments for medical schools and better legislation providing state control of medical practice and licensure.

(*l*) This country should not be satisfied with medical standards unless they are at least equal to those of other world powers which are our competitors in commerce, arts and science.

### A Novel Use for Old Ferry Boats in Treating Consumptives.

By THE COMMITTEE ON THE PREVENTION OF TUBERCULOSIS OF THE CHARITY ORGANIZATON SOCIETY OF THE CITY OF NEW YORK.

THE old Staten Island ferry boat "Southfield" has been cleaned up and moored at the dock at the foot of West 16th street on the North River, where it is now being used as a day camp for consumptives. With a trained nurse in charge, a regular visiting staff of physicians, an abundance of milk and eggs, and steamer chairs and hammocks in which to sit out of doors and watch the passing river craft, fifty men and women are keeping cool and getting back their health and strength.

The boat was put at the disposal of the Committee on the Prevention of Tuberculosis of the Charity Organisation Society of New York, by Commissioner John A. Bensel of the Department of Docks and Ferries. Since the department has been operating its new boats on the Staten Island ferry, the "Southfield" has not been running and the commissioner, therefore, was able to give his hearty support to the plan that was put before him to permit the boat to be used as a day camp under the strict medical supervision of the tuberculosis committee and at the committee's expense. The boat was thoroughly cleaned, water closets, a stove and an ice chest were put in, several dozen steamer chairs and a few cots were bought, a trained nurse was engaged and then the camp was ready for patients.

These patients are sent to the boat after being examined and passed by the physicians in charge of the Associated Tuberculosis Dispensaries to which any one desiring this treatment may go for this purpose. After examination, if the applicant proves to be able to be up and around and is not running a temperature, a card of admission to the boat is given and thereafter, each day the patient goes through the regular routine beginning with the taking of temperatures and weighing at 9 o'clock in the morning and ending at 5 o'clock in the afternoon when all go to their homes except a few men patients who stay all night. Fresh milk and eggs are given in abundance, each patient taking from three to eight eggs and from three to eight glasses of milk daily, other food, except bread and butter, hot tea or coffee and a cooked egg, which are given out at noon, being brought by the patients themselves. Once each week the committee in charge meets on the boat, the medical members of this committee serving each two weeks in turn as visiting physicians. In speaking about the boat a member of the committee said: "A good many people realise now that fresh air and medical oversight are needed to cure tuberculosis, but in a long, narrow, congested place like the island of Manhattan how is this fresh air to be had? There are not parks enough to go around and daily trips to the great open spaces in the Bronx are out of the question for the ordinary sick consumptive who can't take the time or the money to do this.

We looked into this matter carefully, some time ago, several good sites having been very generously offered to us, but we considered them too far from our base of supply, the crowded tenements where tuberculosis is bred. Then some old buildings that the city had condemned were about to be put at our disposal, but we could not get any assurance but that we might be put out right after putting in improvements extensive enough to be expensive to us with our limited resources and so we had to give up that idea. We then thought of the water front and found a mighty helpful ally in Commissioner Bensel and it was due to his interest and broad view of things that we now have our camp in full swing aboard the good boat 'Southfield.' It was something of a job to clean her up and fix things as we needed them, but it was well worth while. If any one doubts it, let him go down and see for himself. The patients are putting on pounds and the color is coming back in whitened cheeks in a most wonderful manner. Now and then a good friend sends us some fruit, magazines or flowers, and with these and the extra diet and good fresh air our patients are getting along famously. There's an idea in all this, too, that's worth giving a good deal of thought to. With all our talk about the impossibility of getting fresh air in our tenement districts, and there is no doubt but what that is all too true, have we not the means ready at hand in our large water front or on our bay to provide resting places where our 40,000 consumptives and our thousands of others needing fresh air can get this absolute essential to cure?"

---

If pulmonary tuberculosis is to be prevented either in the family or at large it must be by the abandonment of the old dominant notion of an inherited diathesis and the substitution therefor of the vital belief in infection. The possibility of infection by cow's milk is too great to warrant its being ignored or held in disdain. Tuberculous cattle are a menace to society, and laws for their detection and death are salutary as has been shown in Denmark, where, thanks to strict enforcement of such provisions, tuberculosis has almost been eradicated from the dairy herds. Every physician who is the regular medical attendant of any family in which there has been a case of pulmonary tuberculosis, should regard himself as personally responsible for the prevention of the disease in other members of the household. This applies in particular to the children, who, by reason of their tender age, are especially liable to infection, and cannot be expected to appreciate the necessity and wisdom of prophylactic measures.—*Babcock on "Diseases of the Lungs."*

